# Editorial: Emerging bioanalytical techniques and therapies for human disease models

**DOI:** 10.3389/fbioe.2024.1453813

**Published:** 2024-09-25

**Authors:** Xuerui Wang, Jinnuo Lu, Yixiao Huang, Xinhao Liu, Guocheng Fang, Chih-Tsung Yang, Zhaobin Guo

**Affiliations:** ^1^ Shanghai Frontiers Science Center of TCM Chemical Biology, Institute of Interdisciplinary Integrative Medicine Research, Shanghai University of Traditional Chinese Medicine, Shanghai, China; ^2^ School of Electrical and Electronics Engineering, Nanyang Technological University, Singapore, Singapore; ^3^ Future Industries Institute, University of South Australia, Adelaide, SA, Australia; ^4^ Key Laboratory of Basic Pharmacology of Ministry of Education, Zunyi Medical University, Zunyi, China; ^5^ State Key Laboratory of Quality Research in Chinese Medicine, University of Macau, Macao, China

**Keywords:** organ-on-chips, diseases modeling, drug screening, biosensing, organoids

Modeling a human disease advances in-depth understanding of its progress and paves the way for effective treatments. With the continuous progress in the convergence of pharmacology, molecular biology, medicine/medical techniques and engineering, models that can faithfully recapitulate various physiological or pathological processes within humans are increasingly in demand (Moroni et al., 2018; Searson, 2023; Zhou et al., 2023). In addition, approaches that can rapidly and accurately monitor the onset and progress of events within the model at cellular, sub-cellular, and molecular levels are equally important during the development of these advanced disease models (Leng et al., 2023; Clarke et al., 2021; Fuchs et al., 2021). The integration of modeling and sensing technologies not only provides robust support for new drug discovery (Guo et al., 2022a), but also lays a solid foundation for the development of personalised therapeutics.

So far, traditional two-dimensional (2D) cell cultures and animal models are still the main approaches to establish human disease models and perform drug screening. However, they fail to provide effective and accurate preclinical assessment of drug efficacy and toxicity (Brancato et al., 2020). Although *in vitro* cell culture in Petri dishes is a simple, high-throughput method for preliminary drug screening and testing, these cellular models usually lack *in vivo* tissue microstructure and physiological functions, resulting in an inability to mimic cellular functions and signaling pathways in tissues (Linville et al., 2022; Guo et al., 2019). In addition, there are significant differences in species between animals and humans, albeit animal experiments are the gold standard for preclinical validation in drug development (Brancato et al., 2020; Jucker, 2010). FDA therefore revoked the requirement of animal tests for new drugs in its recent Modernization Act 2.0 (Wadman, 2023). Additional limitations of animal experiments include the microscopic imaging (Cheng and Cheng, 2021), the presence of confounding variables (Schellinck et al., 2010; Narayan et al., 2021), the costs and the availability (e.g., non-human primates) (Chu et al., 2022), and animal ethics. Therefore, there is an urgent need for alternative tissue models that better mimic human pathophysiology to bridge the gap in disease research as well as in the development of new drugs.

**FIGURE 1:**
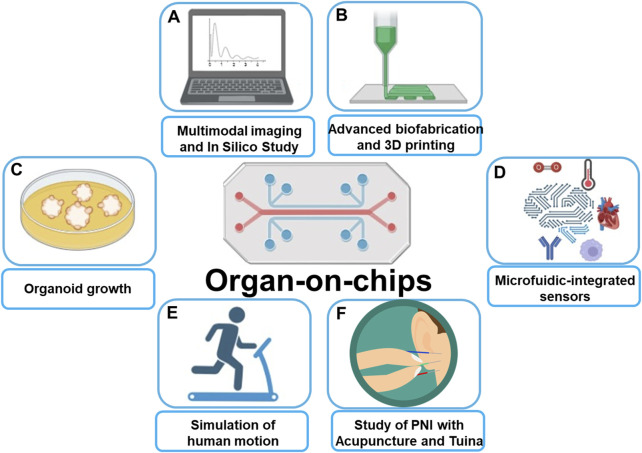
Schematic illustration of Organ-on-chips applications: **(A)** Allow for multimodal imaging and *in silico* study. **(B)** Combining with advanced biofabrication and 3D printing leads to organ-on-chips more precisely mimic real human tissues. **(C)** Presence of shear stress and vascular structures make Organ-on-chips more suited for organoid growth than traditional models. **(D)** Combining with microfluidics technology allows for precise control of temperature, oxygen, shear forces, etc. **(E)** Organ-on-chips mimics human movement for simulation of the influence from physical movements on vascular biology. **(F)** Perspective of using Organ-on-chips to predict the therapeutic effects of acupuncture and Tuina on peripheral nerve injuries.

The recent development of 3D spheroid (Zhang et al., 2022; Zhang et al., 2021; Yan et al., 2023), organoid (Broutier et al., 2017; Tuveson and Clevers, 2019; Beydag-Tasöz et al., 2023), organ-on-a-chip (Guo et al., 2022b; Shin et al., 2024; Del Rio et al., 2023; Fang et al., 2023), and 3D bioprinting (Tang et al., 2020; Tang et al., 2024; Tung et al., 2024) have facilitated the creation of physiological tissue models and complex disease models that are more compatible with physiological and pathological studies. Such preclinical models are critical to more effective translate cancer research into new treatment options for cancer patients. Figure 1 briefly introduces the application scenario of organ-on-chip that covers the Research Topic of papers published in this Research Topic.

In this Research Topic, Zhu et al. summarised and discussed recent advances in 3D modeling for liver cancer research, including the creation of 3D models, traditional 3D cell culture techniques, and microfluidic techniques for constructing “organ-on-chip”. In addition, the application of 3D bioprinting technology to develop of liver cancer organoid and patient-derived xenograft (PDX) models was also discussed (Zhu et al.). They pointed out that 3D models provide realistic and reliable tools for advancing liver cancer research. By simulating tumour heterogeneity and microenvironment, these models contribute to a better understanding of disease mechanisms and provide new strategies for personalised treatment (Zhu et al.). In this momentum, engineered 3D *ex vivo* tissue models have also been developed to significantly boost accurate human disease modelling. Significant advances in tissue engineering, microfabrication techniques, and cell biology have led to the development of organotypic models with higher complexity for mechanobiological studies and therapeutic trials. Advanced model systems also allow *in vivo* reconstruction of fundamental cellular component aspects of organ tissues, as well as natural-like mechanical conditions (i.e., matrix stiffness, shear stress) to maintain cells and engineered tissues in physiologically relevant microenvironments.

Review of the haemodynamic mechanisms behind exercise-induced vascular adaptations by Sun et al. provides a scientific basis for understanding how exercise promotes vascular health. For example, organ-on-a-chip can provide a unique platform for vascular system research by controlling microchannel flow rates while accommodating multiple cell types, realistically simulating blood perfusion and building multiple vascular models based on different pathological conditions. The construction of new models overcomes the limitations of current research models and methods. It is therefore expected to address the shortcomings in individual differences, providing a more comprehensive understanding of the effects of exercise on the vascular system.

Zhu et al. presented recent advances in lung-like organs and organ-on-a-chip and discussed their applications in lung cancer research and drug evaluation. These developments range from realistic simulations and mechanistic probes of lung cancer to the evaluation of chemotherapeutic agents and targeted therapeutic interventions. The ability of the model to mimic the pathological and physiological microenvironment of the lung allows it to complement or replace existing 2D culture and experimental animal models and has the potential to enable personalised lung cancer therapy.

Furthermore, as previously stated, visualization studies in animal models remain one of the major bottlenecks in current scientific research. Significant progress has been made in small animal imaging in recent years, and a series of advanced techniques, including two-photon imaging, two-region near-infrared imaging (NIR-II), and intravital microscope, have been applied to real-time imaging of tumour metastasis in murine models. However, there are various technical constraints in the practical application, including limitations in imaging depth (1–2 mm), dependence on contrast agent labelling, and limited observation time, etc.

This directly leads to the “black box” status in drug discovery and screening. For example, while we might know the final effect of a drug against lung cancer metastasis, it is difficult to clarify which intermediate steps in the metastatic process are effectively inhibited or “off-target” by the drug. To this end, Li et al. designed and synthesized a series of near-infrared (NIR) fluorescent probe substrates to monitor BChE activity, and ultimately selected a NIR fluorescent probe substrate named CYBA. This probe can be selectively metabolized by BChE, showing enhanced infrared fluorescence with high selectivity and sensitivity. This provides a novel, practical, and reliable method to monitor and visualize BChE activity.

To date, Chinese medicine rehabilitation methods have been validated to be effective for therapy of various diseases, but their cellular and molecular mechanisms are still unknown. Liu et al. summarised the molecular mechanisms of Chinese medicine rehabilitation techniques and new biomaterials in the treatment of peripheral nerve injuries and explored the research direction of the combination of the two for precision treatment at both macro and micro levels. By combining Tui Na with new materials and other advanced fabrication techniques including organ-on-chip models, the microenvironment of peripheral nerve injury and regeneration are recapitulated *in vitro*. Implementation of Tui Na with novel organ-on-chip models allows researchers to objectively and quantitatively study the effects of various Tui Na techniques on peripheral nerve regeneration, such as different strengths, angles, frequencies, intervention times, treatment times, etc. The combination of Tui Na and microfluidic technology, as well as imaging or sensing technologies, can significantly advance its application in injury regeneration or pharmacological researches (Ma et al., 2023; Wang et al., 2023; Chen et al., 2023; Liu et al.).

In summary, an advanced human model with high fidelity to the human environment, high-throughput screening, and visualization research is becoming a pressing Research Topic (He et al., 2022; Zhang et al., 2023). Emerging technologies such as 3D bioprinting, organ (organoid)-on-a-chip, biosensing, and imaging are continuously developing (Dai et al., 2022), and are expected to have a profound impact on medical research and clinical practice in the future. However, organ-on-a-chip systems are still not broadly employed in the pharmaceutical industry due to the challenges in addressing the practical standardization for rapid drug discovery and accurate preclinical assessment. In the long term, the continued integration of new concepts and technologies into organ-on-a-chip platforms promises to bridge the biological and technological gap between translational, preclinical, and clinical research. Therefore, we envisage the capability of organ-on-a-chip applications in the pharmaceutical industry and its increasingly bright future in the field of personalized and precision medicine.
